# Impact of Variable RNA-Sequencing Depth on Gene Expression Signatures and Target Compound Robustness: Case Study Examining Brain Tumor (Glioma) Disease Progression

**DOI:** 10.1200/PO.18.00014

**Published:** 2018-09-13

**Authors:** Alexey Stupnikov, Paul G. O’Reilly, Caitriona E. McInerney, Aideen C. Roddy, Philip D. Dunne, Alan Gilmore, Hayley P. Ellis, Tom Flannery, Estelle Healy, Stuart A. McIntosh, Kienan Savage, Kathreena M. Kurian, Frank Emmert-Streib, Kevin M. Prise, Manuel Salto-Tellez, Darragh G. McArt

**Affiliations:** **Alexey Stupnikov**, **Paul G. O’Reilly**, **Caitriona E. McInerney**, **Aideen C. Roddy**, **Philip D. Dunne**, **Alan Gilmore**, **Stuart A. McIntosh**, **Kienan Savage**, **Kevin M. Prise**, **Manuel Salto-Tellez**, and **Darragh G. McArt**, Queen’s University Belfast; **Tom Flannery**, **Estelle Healy**, and **Manuel Salto-Tellez**, Belfast Health and Social Care Trust, Belfast, United Kingdom; **Alexey Stupnikov**, Johns Hopkins University, Baltimore, MD; **Hayley P. Ellis** and **Kathreena M. Kurian**, Brain Tumour Research Centre, University of Bristol, Bristol, United Kingdom; and **Frank Emmert-Streib**, Tampere University of Technology, Tampere, Finland.

## Abstract

**Purpose:**

Gene expression profiling can uncover biologic mechanisms underlying disease and is important in drug development. RNA sequencing (RNA-seq) is routinely used to assess gene expression, but costs remain high. Sample multiplexing reduces RNA-seq costs; however, multiplexed samples have lower cDNA sequencing depth, which can hinder accurate differential gene expression detection. The impact of sequencing depth alteration on RNA-seq–based downstream analyses such as gene expression connectivity mapping is not known, where this method is used to identify potential therapeutic compounds for repurposing.

**Methods:**

In this study, published RNA-seq profiles from patients with brain tumor (glioma) were assembled into two disease progression gene signature contrasts for astrocytoma. Available treatments for glioma have limited effectiveness, rendering this a disease of poor clinical outcome. Gene signatures were subsampled to simulate sequencing alterations and analyzed in connectivity mapping to investigate target compound robustness.

**Results:**

Data loss to gene signatures led to the loss, gain, and consistent identification of significant connections. The most accurate gene signature contrast with consistent patient gene expression profiles was more resilient to data loss and identified robust target compounds. Target compounds lost included candidate compounds of potential clinical utility in glioma (eg, suramin, dasatinib). Lost connections may have been linked to low-abundance genes in the gene signature that closely characterized the disease phenotype. Consistently identified connections may have been related to highly expressed abundant genes that were ever-present in gene signatures, despite data reductions. Potential noise surrounding findings included false-positive connections that were gained as a result of gene signature modification with data loss.

**Conclusion:**

Findings highlight the necessity for gene signature accuracy for connectivity mapping, which should improve the clinical utility of future target compound discoveries.

## INTRODUCTION

Gene expression profiling examines the altering state of the transcriptome at many levels. In cancer research, gene expression profiling has been essential in assessing biologic function, pathogenesis, and biomarker discovery.^1,2^ In the past, microarrays have been used to measure gene expression; however, methodological drawbacks include background hybridization, reliance on established probes, and limited dynamic range.^3-5^ A superior method available for gene expression measurement is RNA sequencing (RNA-seq) of cDNA transcripts in a high-throughput manner. Sequencing reads are then aligned to a reference genome or transcriptome and mapped to an identified region. Transcript abundance is estimated, facilitating the comparison of gene expression profiles. RNA-seq has wider analytical capabilities, including single nucleotide variants, insertion-deletions, gene splice variants, post-transcriptional modifications, and gene fusion detection, but remains costly.^6,7^ Experimental techniques developed to minimize sequencing costs include sample multiplexing. Multiplexing involves labeling each sample library with a barcode identifier, allowing multiple libraries to be pooled and sequenced simultaneously, reducing costs.^7-10^ Smaller volumes of RNA are analyzed for multiplexed samples; thus, the downside to multiplexing is reduced sequencing depth for this library type.

Accurate assessment of transcripts depends on length, abundance, and mappability to the reference and sufficient sequencing depth, particularly for genes with low transcript abundance.^11,12^ Sequencing depth alterations can affect the detection of differentially expressed genes (DEGs) and potentially the accuracy of RNA-seq–based downstream analysis. Few studies have assessed the impact of sequencing depth alterations on RNA-seq downstream applications.^13^ More studies are required, particularly to assess applications that rely on precise gene signatures, informative in classifying cancer subtypes and improved prognostic and predictive outcomes.^14,15^ A gene signature is summarized by DEGs that collectively represent the most prominent features of a cancer subtype or disease progression phenotype. If a gene signature is compiled using gene expression profiles with low sequencing depth, then it may not be fully representative of that phenotype. This could be particularly problematic for connectivity mapping that examines a gene expression signature contrast with the aim of predicting potentially therapeutic US Food and Drug–approved target compounds for repurposing.^16^

There is urgent need for new targeted therapies for gliomas, which are the most common form of primary brain tumor. Gliomas can be classified from grade I to IV on the basis of histologic and molecular information.^17^ Depending on the cell of origin, each neoplasm is classified as an astrocytoma, oligodendroglioma, or ependymoma. Diffuse astrocytoma (WHO grade II) can demonstrate progression to anaplastic astrocytoma (WHO grade III) and malignant glioblastoma (GBM; WHO grade IV). Patient survival beyond 5 years is 58% for grade II astrocytoma, 23.6% for grade III anaplastic astrocytoma, and only 5% for grade IV GBM.^18-20^ Patients with GBM undergo concurrent chemoradiotherapy with temozolomide according to the Stupp protocol and adjuvant chemotherapy.^21^ Patients with anaplastic glioma may undergo radiotherapy with or without chemotherapy, depending on tumor molecular profile.^22^ Low-grade gliomas with poor prognosis may also be considered for adjuvant treatment.^23^ There has been minimal improvement in overall survival (14.6 *v* 12.2 months)^24^; thus, new treatments are urgently sought for glioma. Herein, reference gene signatures were compiled from publically available sequenced tumors for astrocytoma disease progression.^2^ Subsampling was applied to simulate sequencing depth alterations of gene signatures, and the performance of connectivity mapping was assessed. Results reveal that information loss to gene signatures significantly affects target compound robustness.

## METHODS

Published whole transcriptome sequencing data of brain tumor biopsy specimens from adults (accession: GSE48865; Bao et al^2^) was downloaded from the Sequence Read Archive.^25^ On average, samples had 50 million reads each. Reads were quality controlled using Trimmomatic software^26^ and aligned using Bowtie2,^27^ allowing one mismatch against the human genome version *hg38*.^28^ Aligned reads were mapped to genes from the GRCh38.81 annotation^29^ using samExploreR software.^30,31^

**Fig A1.**
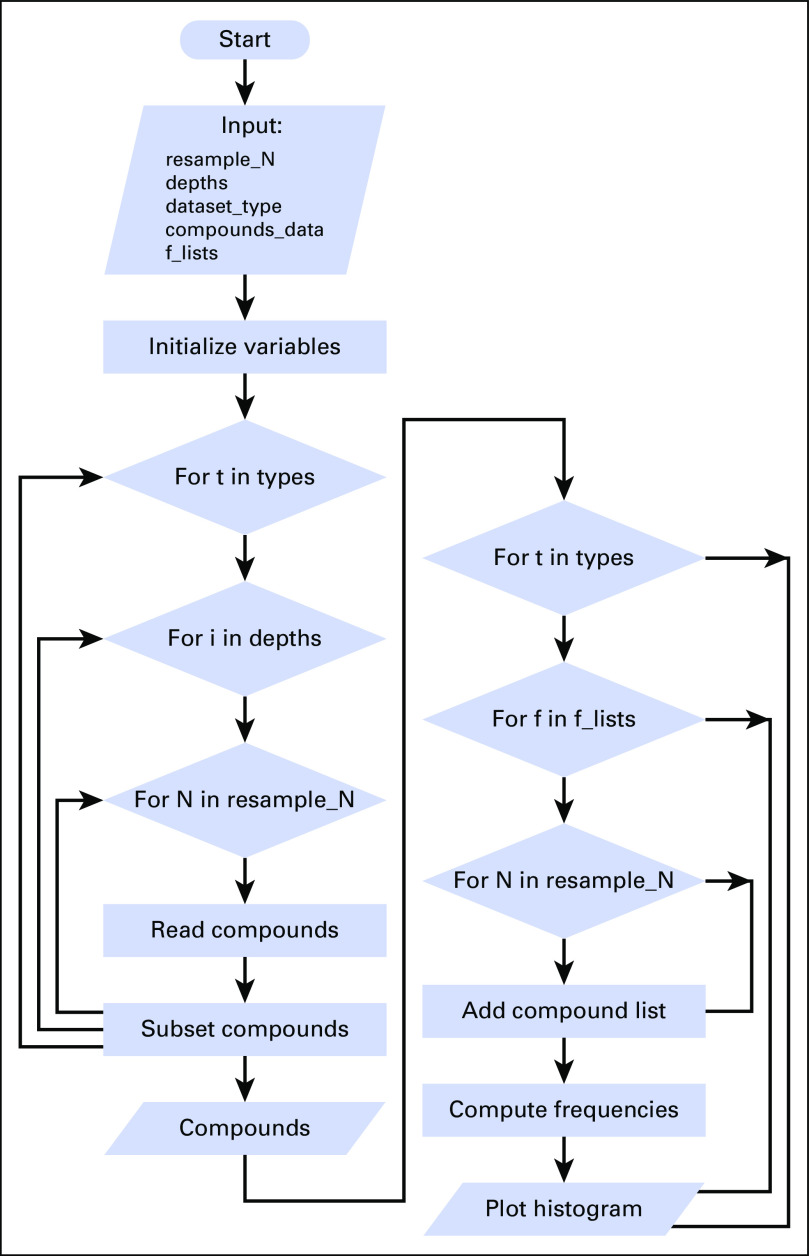
Schematic depicting the bioinformatics pipeline implemented for the subsampling iterative analysis of connectivity mapping for target compound identification. Subsampling using 17 different data fractions (*f* = 0.01 to 1.0) and 25 iterations per data fraction providing 425 data sets with variable sequencing depth per gene signature. Count vectors were analyzed to detect differentially expressed genes (DEGs) and saved as gene lists. Gene lists were subsequently analyzed in connectivity mapping for target compound identification. RNA-seq analysis measured DEG, gene ontology (GO) terms, and target compound identification for the low- to high- (Dataset_I) and the high- to high-grade (Dataset_II) astrocytoma gene signatures.

To benchmark a diverse range in performance of the RNA-seq analysis, mapped reads were subsampled to simulate samples with a range of lower cDNA library sequencing depths using a bioinformatics pipeline^32^ (Appendix Fig A1; Data Supplement). RNA-seq reads for transcript-level abundance to gene level were summarized and normalized using the relative log expression method and analyzed for differential expression using full (*f* = 1.0) and simulated samples with DESeq2.^33^ Gene expression signature contrasts representative of astrocytoma disease progression were compiled for low to high (L-H) and high to high (H-H)–grade astrocytoma (Data Supplement). Gene signature contrasts were assessed for consistency in a heatmap using pheatmap R package (http://CRAN.R-project.org/package=pheatmap). The impact of information loss to gene signatures for DEGs, gene ontology (GO) terms, and target compound detection was assessed using differential expression, GO, and gene expression connectivity mapping analysis, respectively, with DESeq2, GOseq, and the QUB Accelerated Drug and Transcriptomic Connectivity (QUADrATiC) software^33-35^ (Data Supplement). The reproducibility of significant connections to the Library of Integrated Cellular Signatures identified for all cell lines and neuronal specific cell lines (Data Supplement) by QUADrATiC was investigated^16,36-38^ (Data Supplement). Results and associated false discovery rates (FDRs) were visualized using the R packages VennDiagram and ggplot2.^39,40^

## RESULTS

### Assessment of the L-H and H-H Gene Expression Signatures

**Fig 1.**
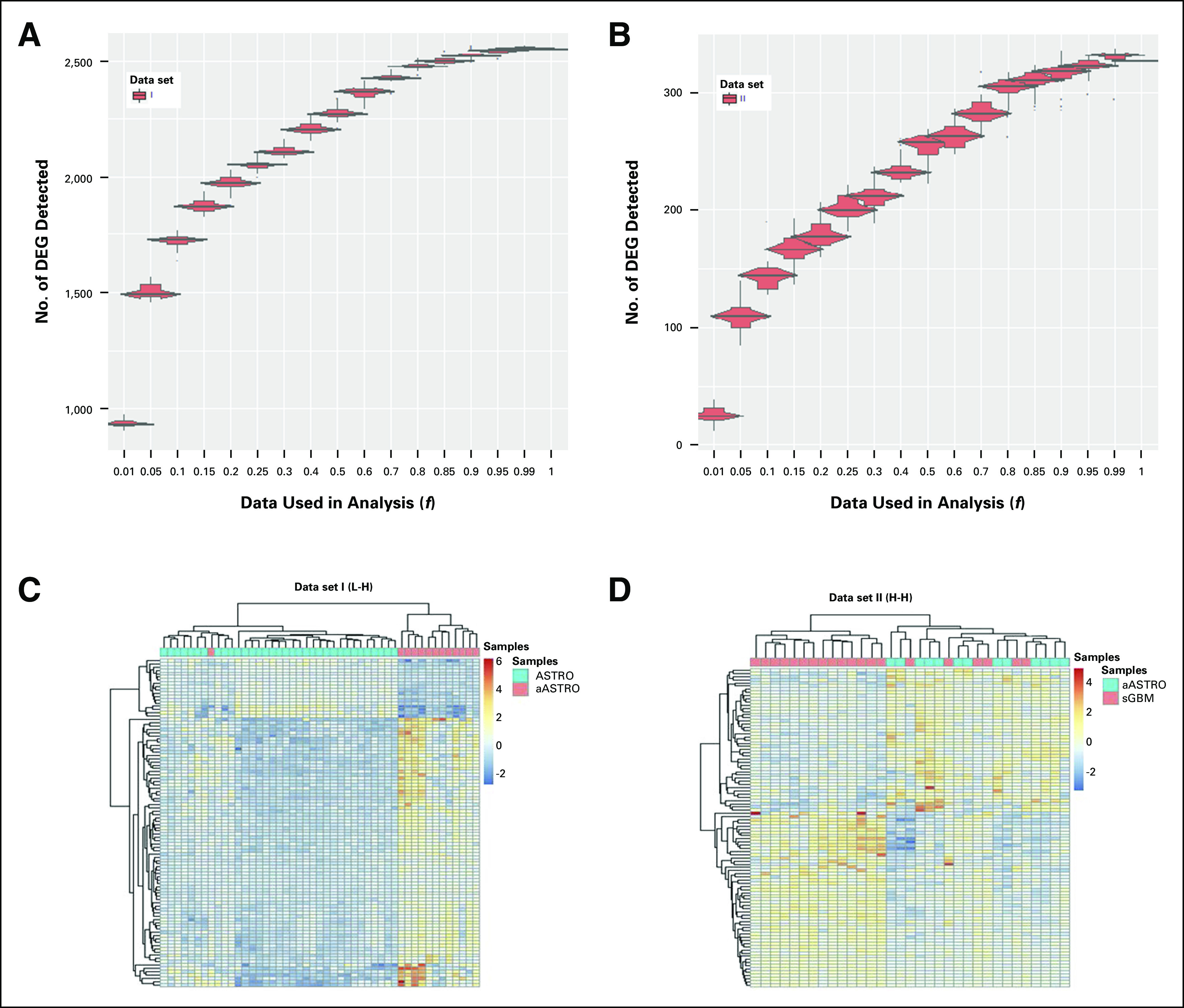
Effect of decreased cDNA library sequencing depth on the number of differentially expressed genes (DEGs) detected from (A) Dataset_I, and (B) Dataset_II gene signatures (Data Supplement). Visualization of the global stratification ability of (C) Dataset_I, low to high (L-H), and (D) Dataset_II, high to high (H-H) gene signatures. Dataset_I is composed of astrocytomas (ASTRO) and anaplastic astrocytomas (aASTRO). Dataset_II is composed of aASTRO and secondary glioblastomas (sGBM). Heatmap was generated using unsupervised hierarchical clustering with the full RNA-seq data (*f* = 1) and depicts the gene expressional patterns of the top 100 differentially expressed genes identified between the gene signature contrast groups. The WHO disease grades of samples as determined by Bao et al^2^ are overlaid.

L-H (Dataset_I) and H-H (Dataset_II) gene signature contrasts comprised 47 and 33 patients, respectively (Data Supplement). Some 6,648 DEGs were identified for Dataset_I, which reduced to 2,550 after filtering (Fig 1A). Just 608 DEGs were identified for Dataset_II, reducing to 327 after filtering (Fig 1B). Each gene signature contrast clustered into two separate branches, which mostly stratified patients on the basis of disease grade (Figs 1C and 1D). Dataset_I outperformed Dataset_II; all but one patient clustered according to disease grade. For each gene signature contrast, no outliers outside of the two disease grades were identified.

### Impact of Information Loss to Gene Signatures for DEG and GO Detection

**Fig A2.**
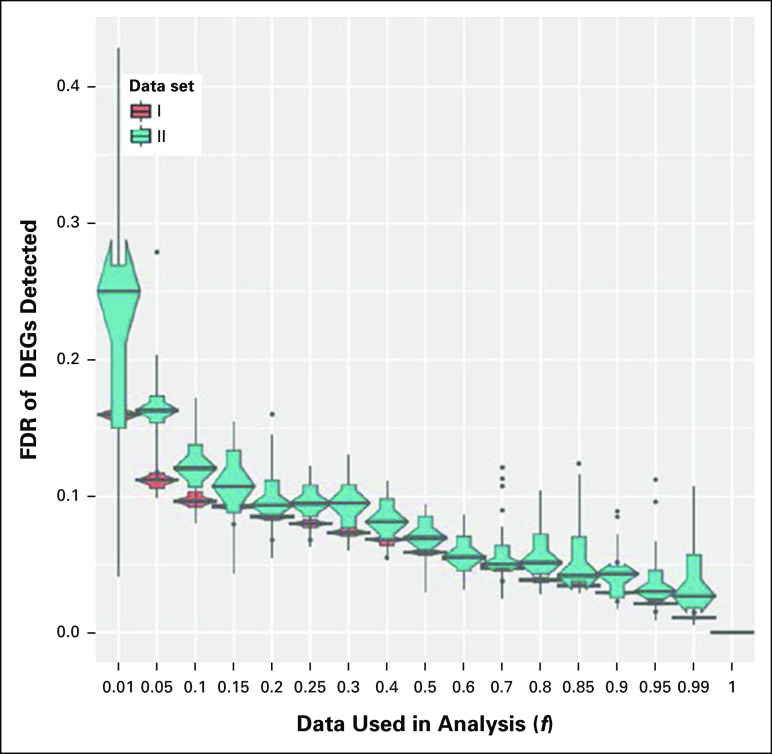
Effect of decreased cDNA library sequencing depth on the false discovery rate (FDR) for the number of differentially expressed genes (DEGs) detected for Dataset­_I and Dataset_II gene signatures.

For Dataset_I, initial reductions in data analyzed (*f* = 0.8 to 1.0) did not greatly affect the number of DEGs detected (Fig 1A). However, the rate of loss of DEGs increased after *f* = 0.8. For Dataset_II, data loss was immediate, and DEG detection reduced equally for every fraction analyzed, as indicated by the linear relationship (Fig 1B). Variation in the number of DEGs detected was lower for Dataset_I compared with Dataset_II, as evidenced by smaller confidence intervals. When data input was reduced, the FDR for the number of DEGs detected increased linearly and by approximately the same amount for both data sets (Appendix Fig A2). Dataset_I gene signature therefore demonstrated better resilience to data loss for DEG detection compared with Dataset_II.

**Fig A3.**
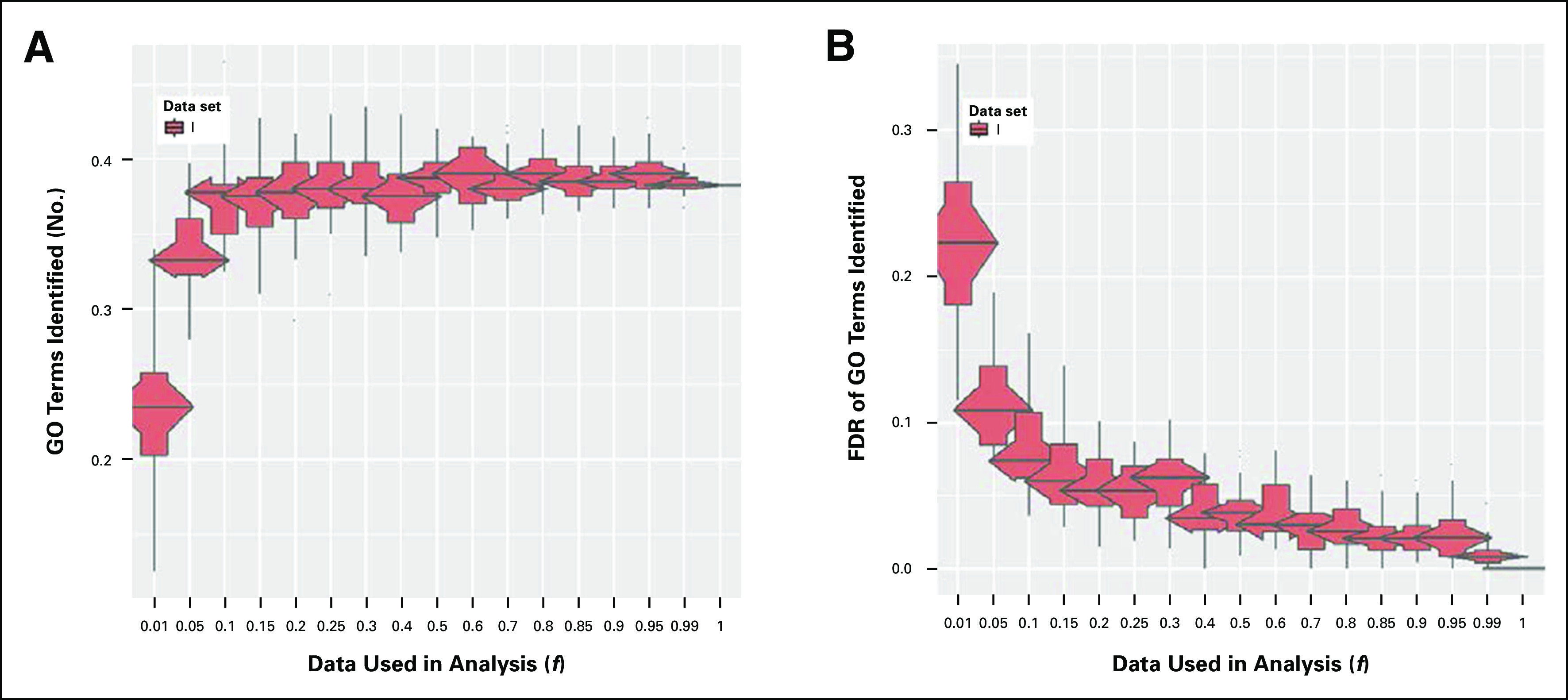
Effect of decreased cDNA library sequencing depth on the number of gene ontology (GO) terms identified for (A) Dataset_I and (B) the false discovery rate (FDR) for the number of GO terms identified. GO analysis for Dataset_II identified just three pathways; therefore, results are not shown.

For the full data set (*f* = 1.0), > 200 GO terms described the functions of the DEGs identified for Dataset_I (Appendix Fig A3A). Thus, heterogeneous biologic functions are involved in low- to high-grade astrocytoma disease transition. For Dataset_I, only small decreases in GO terms were detected using data fractions between *f* = 1.0 and 0.1 (Appendix Fig A3A). Thus, GO term detection was more stable compared with DEGs when Dataset_I gene signature had data loss. The impact of data loss on FDR for GO term detection was on the same scale as that observed for DEG detection for Dataset_I (Fig A3B). Comparatively fewer GO terms, just three, described the DEGs in Dataset_II for the full data set. Given this low number, which reduced to zero on *f* = 0.5, GO results for subsampled Dataset_II are not depicted.

### Impact of Information Loss to Gene Signatures Used in Gene Expression Connectivity Mapping

**Fig 2.**
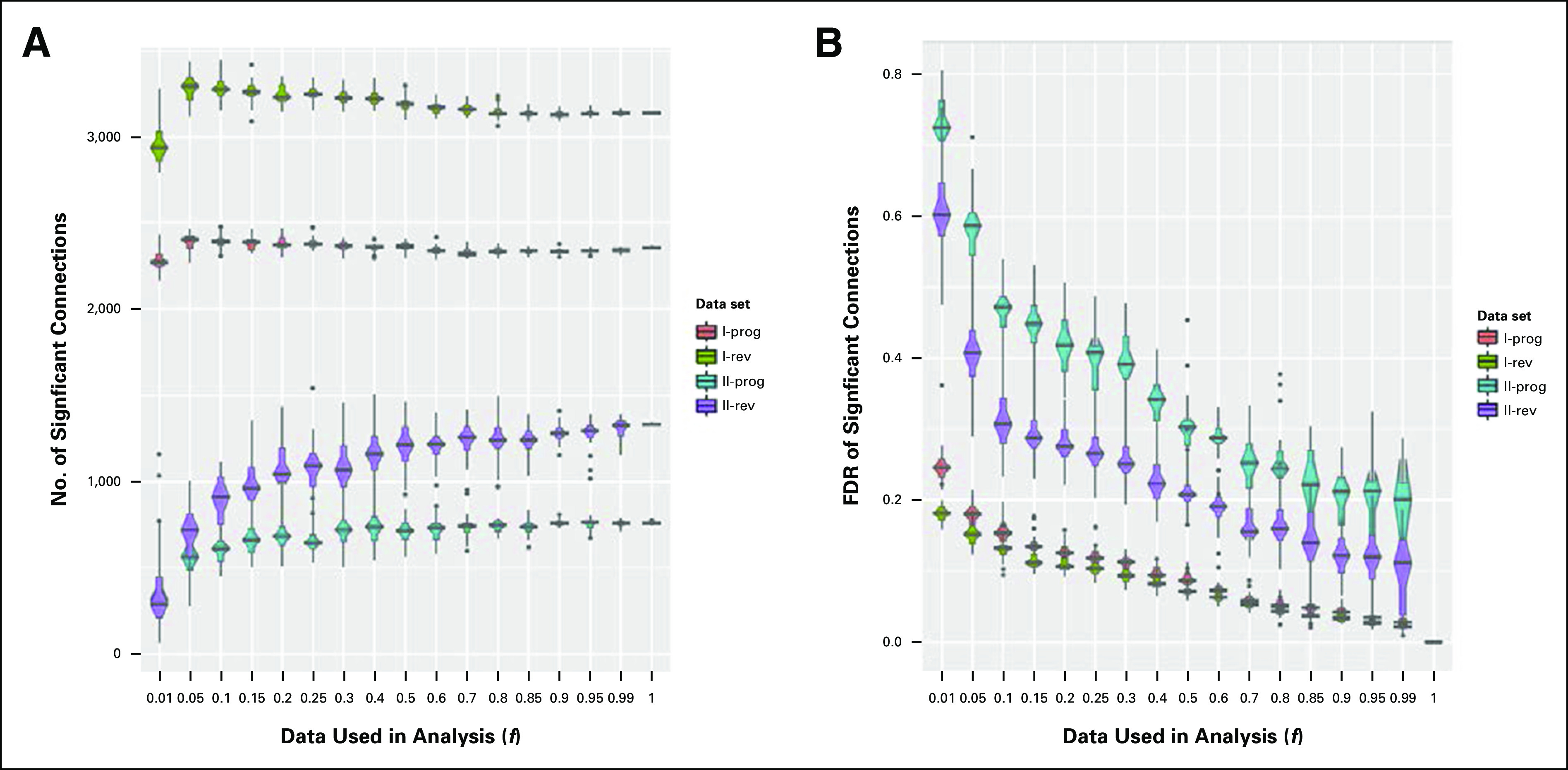
(A) Effect of decreased cDNA library sequencing depth on the number of significant connections detected by connectivity mapping for Dataset_I and II gene signatures. (B) False discovery rate (FDR) of the number of significant connections detected in the connectivity mapping for Dataset_I and II gene signatures. Significant connections that potentially could progress (prog) or reverse (rev) the disease phenotype and FDRs are plotted against the data fraction included in the analysis (*f*).

For the full data set, a greater number of significant reverse (rev) and progress (prog) connections were identified for Dataset_I compared with Dataset_II (Fig 2A). For Dataset_I, data loss did not greatly affect the number of significant rev and prog connections detected. With increasing data loss, Dataset_I significant connections remained relatively stable (*f* = 1.0 to 0.7) and then slightly increased. For Dataset_II, rev significant connections decreased steadily with data loss, whereas prog connections were slightly more stable. Dataset_I displayed less variability in the number of significant connections identified, compared with Dataset_II, as evidenced by smaller confidence intervals. For both Dataset_I and Dataset_II, FDR for the number of significant connections increased steadily with decreasing data used (Fig 2B). However, FDR was three-fold greater for Dataset_II and quickly increased to approximately 10% and 20% for rev and prog connections, respectively, when just 1% of reads were removed (*f* = 0.99). For target compound identification, Dataset_I was therefore more resilient to alterations in cDNA sequencing depth compared with Dataset_II.

**Fig 3.**
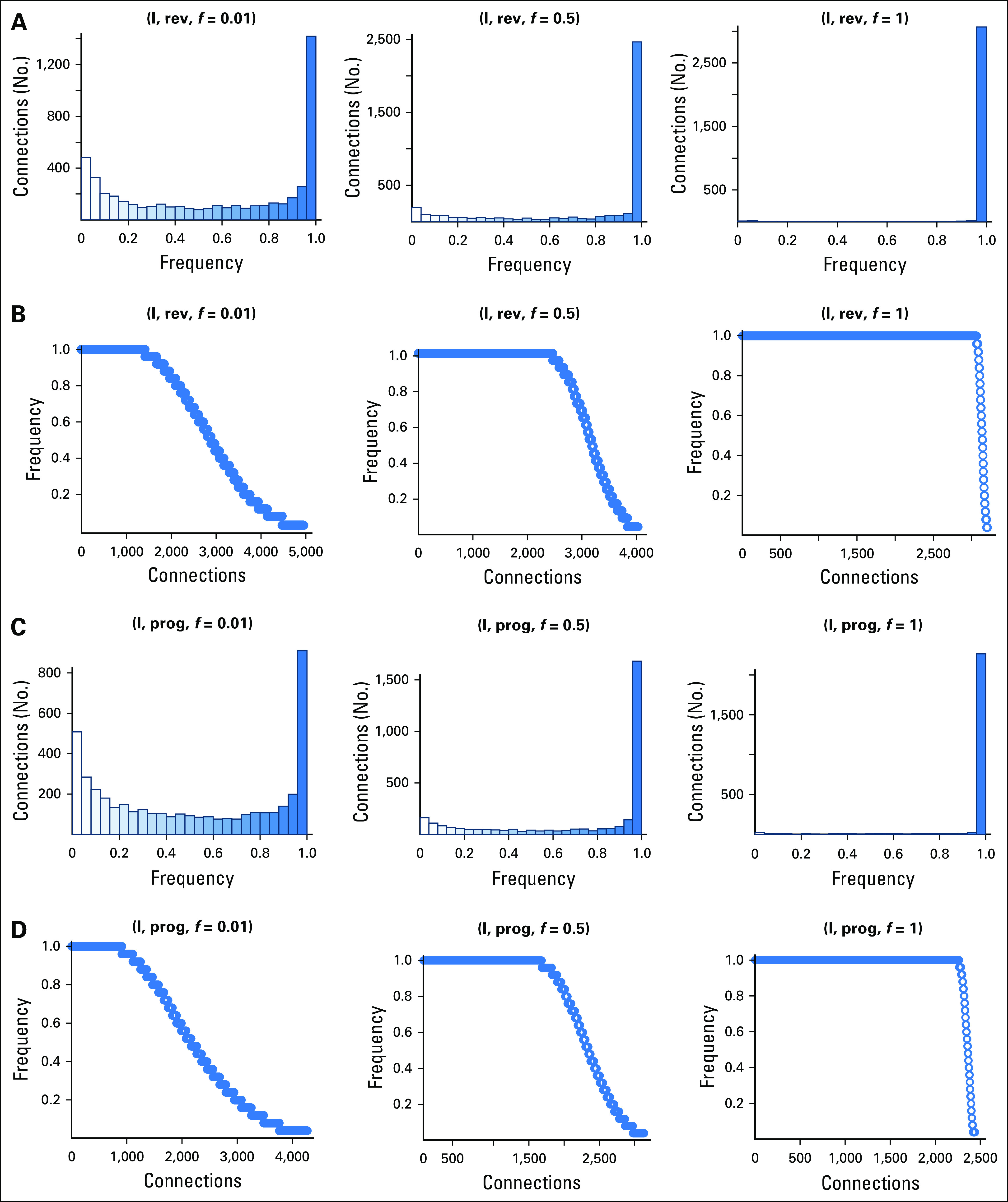
Frequency of progress (prog) and reverse (rev) significant connections to target compounds identified for Dataset_I and II gene signatures. Results for three different subsampled data fractions (*f* = 0.01, 0.5, 1) each with 25 iterations are presented.

**Fig 4.**
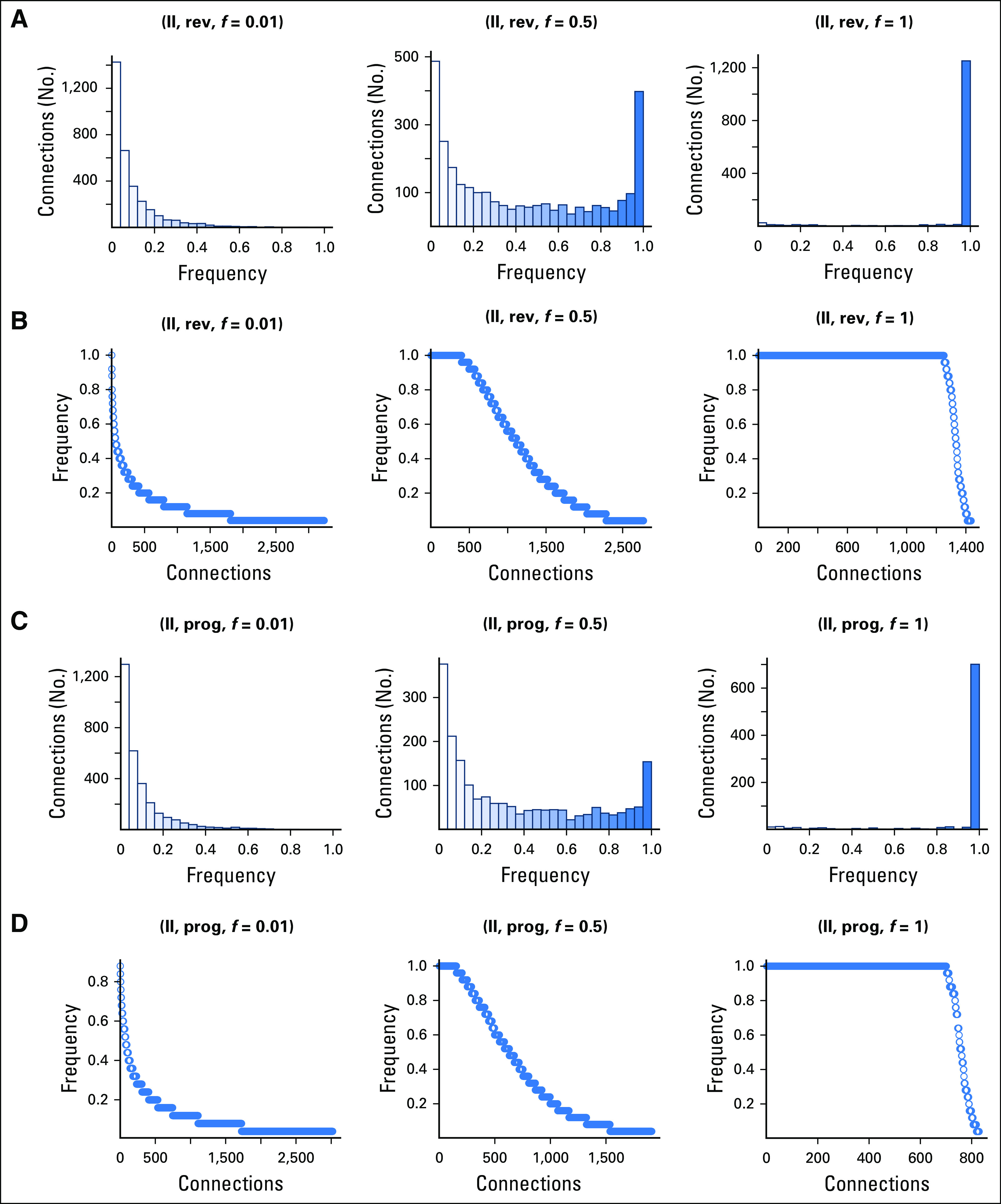
Frequency of progress (prog) and reverse (rev) significant connections to target compounds identified for Dataset_II gene signatures. Results for three different subsampled data fractions (*f* = 0.01, 0.5, 1) each with 25 iterations are presented.

**Fig 5.**
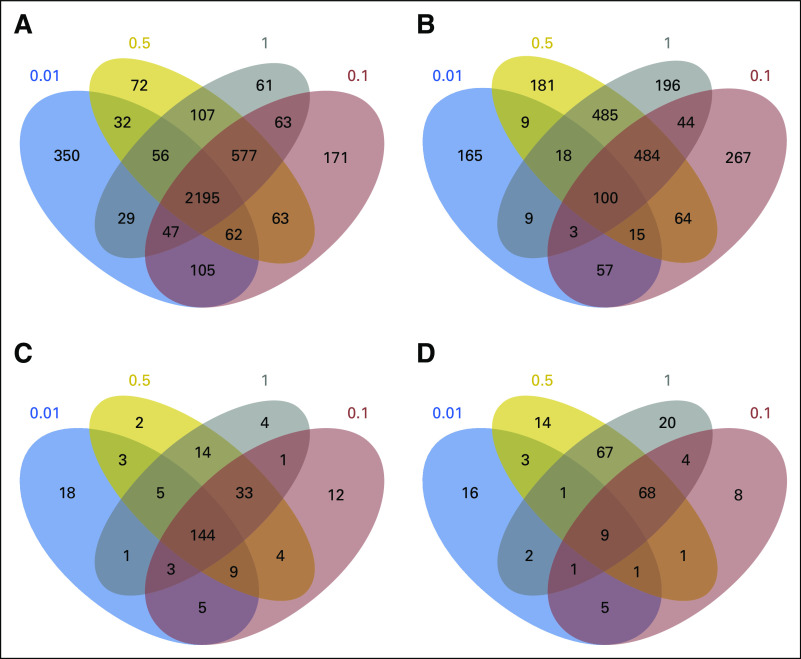
Effect of decreased cDNA library sequencing depth on the number of significant connections to target compounds identified that could potentially reverse the disease phenotype. Significant connections to target compounds identified across all cell lines using (A) Dataset_I and (B) Dataset_II gene signatures with subsampling (0.01, 0.1, 0.5, 1) are illustrated in the Venn diagrams. Significant connections to target compounds identified from the neuronal derived cell lines using (C) Dataset_I and (D) Dataset_II across gene signatures are also compared.

The impact of data loss to gene signatures and the reproducibility of connectivity mapping is presented in Figures 3-5. When full data sets were used for the gene signature (*f* = 1.0), target compound identification was consistent, and mostly the same compounds were identified between iterations (Figs 3, 4A, and 4C; frequency = 1.0). With data loss to the gene signature (*f* = 0.01, 0.5), fewer compounds were consistently identified, and a higher number of target compounds were detected at low frequencies of iterations. For example, 3,135 rev connections were identified for Dataset_I using *f* = 1.0; this increased to approximately 5,000 when subsampled to *f* = 0.01, but approximately 60% of compounds were infrequently detected (Figs 3B and 3C). Proportion of significant connections that are consistently identified decreases with data loss, but the impact was less for Dataset_I. For Dataset_I, when 50% of reads were removed, approximately 62.5% rev (approximately 2,500 of 4,000) and approximately 50% prog significant connections (approximately 1,500 of 3,000) were identified with every iteration (Fig 3). For Dataset_II, when 50% of reads were removed, just approximately 13% rev (approximately 400 of 3,000) and 9% prog significant connections (approximately 180 of 2,000) were identified with every iteration (Fig 4). No robust calls were identified for Dataset_II at *f* = 0.01, and little improvement was observed when half the reads were included (*f* = 0.5; Fig 4). Gene signatures differed in the proportion of significant connections that were consistently identified when cDNA sequencing depth was reduced. When affected by data loss, connectivity mapping results were more robust for Dataset_I compared with the Dataset_II gene signature.

Reducing data to the gene signature led to the loss, gain, and consistent identification of significant connections to target compounds (Fig 5). Compounds consistently identified between data fractions can be seen within the Venn diagram intersections. For Dataset_I, a large proportion of the significant connections across all cell lines (69%; 2,195 of 3,135) and neuronal-specific cell lines (70%; 144 of 205) were detected with all data fractions. Similarly for Dataset_II, a proportion of the significant connections across all cell lines (7%; 100 of 1,339) and neuronal-specific cell lines (5%; nine of 172) were detected with all data fractions. The gain in significant connections can be seen in the relative complement sections of the smaller data fractions in the Venn diagrams. For example, 350 and 105 compounds were detected across all cell lines for Dataset_I, *f* = 0.01, that were not identified by the full data set (Fig 5A). These connections were false positives, because they had not been detected with the full gene signature. Last, we examined the loss of significant connections to target compounds for Dataset_I and II. When 50% of the reads were removed, nine and 27 target compounds identified for neuronal-specific cell lines were lost, respectively, for Dataset_I and II (Figs 5C and 5D; Table 1). Thus, for Dataset_II, more target compounds identified by the full gene signature were lost, and some of these included compounds of potential clinical utility for glioma, such as suramin and dasatinib (Table 1). A comparison of the rate of impact of data loss on GO terms and significant connections detection for Dataset_I can be seen by comparing Figures A3 and 2A.

**Table 1.**
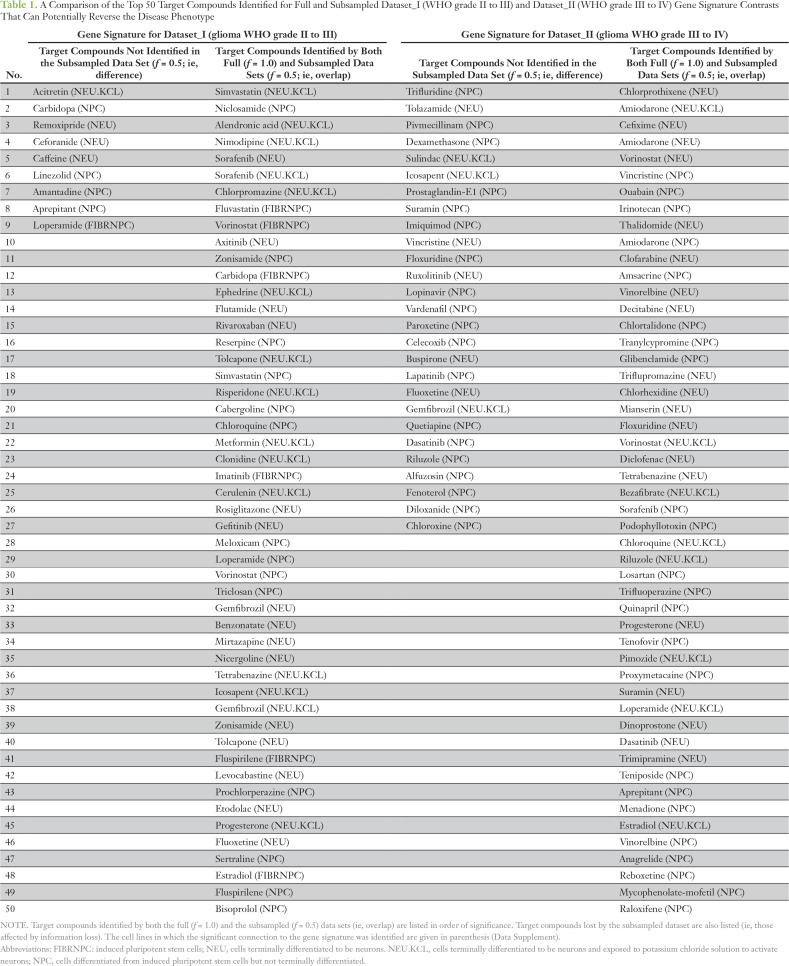
A Comparison of the Top 50 Target Compounds Identified for Full and Subsampled Dataset_I (WHO grade II to III) and Dataset_II (WHO grade III to IV) Gene Signature Contrasts That Can Potentially Reverse the Disease Phenotype

## DISCUSSION

Understanding molecular pathways and regulatory networks driving cancer is essential for the development of new therapies. Gene expression profiling using RNA-seq has led to the development of clinically relevant gene signatures that are informative for cancer subtypes.^14,15^ RNA-seq experimental approaches such as sample multiplexing reduce cDNA sequencing depth and potentially affect gene signature accuracy. This information loss may mask the true biologic variability of a gene signature. Herein, sequence depth alterations in gene signatures were simulated and the impacts of data loss for gene expression connectivity mapping investigated. Two gene signature contrasts representing astrocytoma disease progression were analyzed. Assessment of their global stratification ability revealed that the WHO grade II to III contrast (L-H; Dataset_I) outperformed the WHO grade III to IV contrast (H-H; Dataset_II), whereby more patient gene expression profiles matched their WHO grade classifications. Results support the subjective nature of tumor classification, which has interobserver variability.^41^ Gene signatures provided a framework to assess connectivity mapping output for a well-performing accurate versus a poorer-performing less accurate contrast.

Characterization of the disease progression gene signatures revealed they differed in biologic complexity. L-H gene signature had ten-fold more DEGs (approximately 2,550) compared with the H-H gene signature (327). Results demonstrated the possibility that more genes are involved in low- to high-grade astrocytoma disease transition. After data reduction, DEG loss was not immediate for the L-H gene signature, but with lowering fractions DEG loss increased. For the H-H gene signature, there was immediate and steady DEG loss with reduced data input. FDR for DEG detection increased linearly for both gene signatures; however, the range of FDR values was lower for the L-H gene signature. Thus, the L-H gene signature was more resilient to data loss for DEG detection and had greater test sensitivity compared with the H-H gene signature. Gene signatures also differed in their resilience to data loss for the detection of significant connections to target compounds. Overall, the number of significant connections detected for the L-H gene signature was greater, most likely explained by the heterogeneous biologic mechanisms involved in low- to high-grade astrocytoma transition. With data loss, both rev and prog significant connections remained relatively stable for the L-H gene signature. Data loss led to a steady decrease in rev significant connections for the H-H gene signature; however, prog connections were initially more stable. For both gene signatures, the FDR of significant connections increased with data loss. Overall FDR values and CIs were smaller for the L-H gene signature. For comparative purposes, consider an FDR of 0.1 as an acceptable threshold, where one in every 10 significant connections is a false positive. With data loss, this FDR threshold was reached by the L-H and H-H gene signatures, respectively, when 70% and just 1% of reads were removed. Thus, the L-H gene signature was more resilient to data loss for the detection of significant connections to target compounds using connectivity mapping.

Subsampling of gene signatures for connectivity mapping revealed that the suite of significant connections to target compounds became modified with data loss. Notably, some connections to target compounds of potential clinical utility were lost when the reads were reduced to 50%. Compounds lost by the H-H gene signature (WHO grade III to IV) included suramin, lopinavir, dasatinib, and vincristine, which have already been considered as glioma treatments. Suramin, an anticancer agent, inhibits the binding of growth factors understood to play a role in glioma progression, angiogenesis, and radioresistance and has been used to treat newly diagnosed GBMs.^42,43^ Lopinavir, a protease inhibitor, has reached phase II clinical trials for the treatment of high-grade glioma.^44^ Dasatinib, a kinase inhibitor that acts on members of the Src family of kinases, is well studied in glioma and has shown preclinical promise.^45^ Vincristine, a spindle poison, is used in combination with procarbazine and lomustine to treat high-grade glioma and has also been successful in a phase III trial for the treatment of low-grade gliomas.^22,46,47^ Reductions in transcript abundance probably led to the loss of low-abundance genes from the full gene signature and altered the DEGs detected, leading to the loss of these connectivity mapping connections. Perhaps low-abundance genes that closely characterize the disease phenotype may offer the greatest potential for target compound discovery. If this is the case, then the subsampling approach described herein could potentially identify these important links to target compounds. Fewer significant connections identified by the full data sets were lost by the L-H gene signature compared with the H-H gene signature, suggesting it was more resilient to data loss. It was interesting to note that reduction in cDNA sequencing depth of gene signatures also led to the gain of significant connections to target compounds. Indeed, more significant connections were identified when fewer data were used for both gene signatures; however, few of these connections were consistently identified between iterations. A greater proportion of significant connections were consistently identified with all iterations for the L-H gene signature compared with the H-H gene signature. For connections that were consistently identified, these may have related to the most highly expressed and abundant DEGs in the gene signature contrast. Similarly, in another subsampling RNA-seq study of healthy organisms from multiple taxa, highly expressed genes regulating metabolism and pathogenesis of disease were consistently identified even when downsampling RNA-seq reads to only 1 million reads,^13^ thereby corroborating our findings from diseased tumors.

Results highlight the need for determining the optimal cDNA sequencing depth for accurately identifying DEGs when compiling gene signatures. In the future, RNA standard and spike-in controls may be useful to inform RNA-seq best practices.^48^ The accuracy of a gene signature was particularly important when carrying out additional downstream analyses, such as connectivity mapping. Information loss to gene signatures led to erroneous and false target compound discoveries. Gene signatures with consistent sample classification and gene expression profiles were more resilient to data loss and provided robust target compound discoveries. Given the instability of gene expression, perhaps using ontology types or ontotypes^49^ to characterize contrast phenotypes may be a more reliable approach compared with gene lists in connectivity mapping. Herein, we demonstrate the utility of QUADrATiC software at identifying US Food and Drug Administration–approved compounds that can be repurposed for glioma. Stringent filtering of connectivity mapping results is required to identify reliable significant connections. Subsampling revealed that the connections that were sensitive to data loss were linked to target compounds of potential clinical utility in glioma. These connections may have the best clinical promise for drug repurposing. Other target compounds sensitive to data loss are being tested for their biologic efficacy against glioma stem cells using clonogenic cell survival assays and Western blot analyses in ongoing studies by this research group. For the wider identification of potential therapeutic compounds for repurposing in glioma, gene signatures for oligodendroglioma and ependymoma disease progression could be analyzed using connectivity mapping in the future.

## References

[1] Bai H Harmancı AS Erson-Omay EZ et al Integrated genomic characterization of IDH1-mutant glioma malignant progression Nat Genet 48 59 66 2016 26618343 10.1038/ng.3457 PMC4829945

[2] Bao ZS Chen HM Yang MY et al RNA-seq of 272 gliomas revealed a novel, recurrent PTPRZ1-MET fusion transcript in secondary glioblastomas Genome Res 24 1765 1773 2014 25135958 10.1101/gr.165126.113 PMC4216918

[3] Verhaak RG Hoadley KA Purdom E et al Integrated genomic analysis identifies clinically relevant subtypes of glioblastoma characterized by abnormalities in PDGFRA, IDH1, EGFR, and NF1 Cancer Cell 17 98 110 2010 20129251 10.1016/j.ccr.2009.12.020 PMC2818769

[4] Laffaire J Everhard S Idbaih A et al Methylation profiling identifies 2 groups of gliomas according to their tumorigenesis Neuro-oncol 13 84 98 2011 20926426 10.1093/neuonc/noq110 PMC3018904

[5] Zhao S Fung-Leung WP Bittner A et al Comparison of RNA-Seq and microarray in transcriptome profiling of activated T cells PLoS One 9 e78644 2014 24454679 10.1371/journal.pone.0078644 PMC3894192

[6] Metzker ML Sequencing technologies—The next generation Nat Rev Genet 11 31 46 2010 19997069 10.1038/nrg2626

[7] Hou Z Jiang P Swanson SA et al A cost-effective RNA sequencing protocol for large-scale gene expression studies Sci Rep 5 9570 2015 25831155 10.1038/srep09570 PMC4381617

[8] Smith AM Heisler LE St Onge RP et al Highly-multiplexed barcode sequencing: An efficient method for parallel analysis of pooled samples Nucleic Acids Res 38 e142 2010 20460461 10.1093/nar/gkq368 PMC2910071

[9] Islam S Kjällquist U Moliner A et al Characterization of the single-cell transcriptional landscape by highly multiplex RNA-seq Genome Res 21 1160 1167 2011 21543516 10.1101/gr.110882.110 PMC3129258

[10] Wang L Si Y Dedow LK et al A low-cost library construction protocol and data analysis pipeline for Illumina-based strand-specific multiplex RNA-seq PLoS One 6 e26426 2011 Erratum: PLos One 6:10.1371/annotation/e5ef7afc-7e81-4053-8670-1bb3402f63fd 22039485 10.1371/journal.pone.0026426 PMC3198403

[11] Sims D Sudbery I Ilott NE et al Sequencing depth and coverage: Key considerations in genomic analyses Nat Rev Genet 15 121 132 2014 24434847 10.1038/nrg3642

[12] Liu Y Zhou J White KP RNA-seq differential expression studies: More sequence or more replication? Bioinformatics 30 301 304 2014 24319002 10.1093/bioinformatics/btt688 PMC3904521

[13] Lei R Ye K Gu Z et al Diminishing returns in next-generation sequencing (NGS) transcriptome data Gene 557 82 87 2015 25497830 10.1016/j.gene.2014.12.013

[14] Parker JS Mullins M Cheang MC et al Supervised risk predictor of breast cancer based on intrinsic subtypes J Clin Oncol 27 1160 1167 2009 19204204 10.1200/JCO.2008.18.1370 PMC2667820

[15] Turkington RC Hill LA McManus D et al Association of a DNA damage response deficiency (DDRD) assay and prognosis in early-stage esophageal adenocarcinoma J Clin Oncol 32 2014 suppl; abstr 4015

[16] Lamb J Crawford ED Peck D et al The Connectivity Map: Using gene-expression signatures to connect small molecules, genes, and disease Science 313 1929 1935 2006 17008526 10.1126/science.1132939

[17] Louis DN Ohgaki H Wiestler OD et al WHO Classification of Tumours of the Central Nervous Systemed 4 Lyon, France IARC Press 2016

[18] Okamoto Y Di Patre PL Burkhard C et al Population-based study on incidence, survival rates, and genetic alterations of low-grade diffuse astrocytomas and oligodendrogliomas Acta Neuropathol 108 49 56 2004 15118874 10.1007/s00401-004-0861-z

[19] Smoll NR Hamilton B Incidence and relative survival of anaplastic astrocytomas Neuro-oncol 16 1400 1407 2014 24723565 10.1093/neuonc/nou053 PMC4165416

[20] Brennan CW Verhaak RG McKenna A et al The somatic genomic landscape of glioblastoma Cell 155 462 477 2013 Erratum: Cell 157:753, 2014 24120142 10.1016/j.cell.2013.09.034 PMC3910500

[21] Wen PY Kesari S Malignant gliomas in adults N Engl J Med 359 492 507 2008 18669428 10.1056/NEJMra0708126

[22] Soffietti R Bertero L Pinessi L et al Pharmacologic therapies for malignant glioma: A guide for clinicians CNS Drugs 28 1127 1137 2014 25403944 10.1007/s40263-014-0215-x

[23] Ziu M Kalkanis SN Gilbert M et al The role of initial chemotherapy for the treatment of adults with diffuse low grade glioma: A systematic review and evidence-based clinical practice guideline J Neurooncol 125 585 607 2015 26530261 10.1007/s11060-015-1931-x

[24] Stupp R Hegi ME Mason WP et al Effects of radiotherapy with concomitant and adjuvant temozolomide versus radiotherapy alone on survival in glioblastoma in a randomised phase III study: 5-year analysis of the EORTC-NCIC trial Lancet Oncol 10 459 466 2009 19269895 10.1016/S1470-2045(09)70025-7

[25] Leinonen R Sugawara H Shumway M The sequence read archive Nucleic Acids Res 39 D19 21 2010 suppl 1 21062823 10.1093/nar/gkq1019 PMC3013647

[26] Bolger AM Lohse M Usadel B Trimmomatic: A flexible trimmer for Illumina sequence data Bioinformatics 30 2114 2120 2014 24695404 10.1093/bioinformatics/btu170 PMC4103590

[27] Langmead B Salzberg SL Fast gapped-read alignment with Bowtie 2 Nat Methods 9 357 359 2012 22388286 10.1038/nmeth.1923 PMC3322381

[28] Karolchik D Barber GP Casper J et al The UCSC Genome Browser database: 2014 update Nucleic Acids Res 42 D764 D770 2014 24270787 10.1093/nar/gkt1168 PMC3964947

[29] Flicek P Amode MR Barrell D et al Ensembl 2014 Nucleic Acids Res 42 D749 D755 2014 24316576 10.1093/nar/gkt1196 PMC3964975

[30] Stupnikov A Tripathi S de Matos Simoes R et al samExploreR: Exploring reproducibility and robustness of RNA-seq results based on SAM files Bioinformatics 32 3345 3347 2016 27402900 10.1093/bioinformatics/btw475

[31] Liao Y Smyth GK Shi W featureCounts: An efficient general purpose program for assigning sequence reads to genomic features Bioinformatics 30 923 930 2014 24227677 10.1093/bioinformatics/btt656

[32] Stupnikov A Glazko GV Emmert-Streib F Effects of subsampling on characteristics of RNA-seq data from triple-negative breast cancer patients Chin J Cancer 34 427 438 2015 26253000 10.1186/s40880-015-0040-8 PMC4593382

[33] Love MI Huber W Anders S Moderated estimation of fold change and dispersion for RNA-seq data with DESeq2 Genome Biol 15 550 2014 25516281 10.1186/s13059-014-0550-8 PMC4302049

[34] Young MD Wakefield MJ Smyth GK et al Gene ontology analysis for RNA-seq: Accounting for selection bias Genome Biol 11 R14 2010 20132535 10.1186/gb-2010-11-2-r14 PMC2872874

[35] O’Reilly PG Wen Q Bankhead P et al QUADrATiC: Scalable gene expression connectivity mapping for repurposing FDA-approved therapeutics BMC Bioinformatics 17 198 2016 27143038 10.1186/s12859-016-1062-1 PMC4855472

[36] Lamb J The Connectivity Map: A new tool for biomedical research Nat Rev Cancer 7 54 60 2007 17186018 10.1038/nrc2044

[37] Musa A Ghoraie LS Zhang SD et al A review of connectivity map and computational approaches in pharmacogenomics Brief Bioinform 18 903 2017 28334173 10.1093/bib/bbx023 PMC6113891

[38] McArt DG Dunne PD Blayney JK et al Connectivity mapping for candidate therapeutics identification using next generation sequencing rna-seq data PLoS One 8 e66902 2013 23840550 10.1371/journal.pone.0066902 PMC3694114

[39] Chen H Boutros PC VennDiagram: A package for the generation of highly-customizable Venn and Euler diagrams in R BMC Bioinformatics 12 35 2011 21269502 10.1186/1471-2105-12-35 PMC3041657

[40] Wickham Hggplot2: Elegant Graphics for Data Analysis New York, NY Springer-Verlag 2009

[41] Delattre JY Improving diagnosis and management of primary brain tumors Curr Opin Neurol 30 639 642 2017 28906269 10.1097/WCO.0000000000000500

[42] Laterra JJ Grossman SA Carson KA et al Suramin and radiotherapy in newly diagnosed glioblastoma: Phase 2 NABTT CNS Consortium study Neuro-oncol 6 15 20 2004 14769135 10.1215/S1152851703000127 PMC1871972

[43] Takano S Gately S Engelhard H et al Suramin inhibits glioma cell proliferation in vitro and in the brain J Neurooncol 21 189 201 1994 7699415 10.1007/BF01063768

[44] Ahluwalia MS Patton C Stevens G et al Phase II trial of ritonavir/lopinavir in patients with progressive or recurrent high-grade gliomas J Neurooncol 102 317 321 2011 20683757 10.1007/s11060-010-0325-3

[45] Schiff D Sarkaria J Dasatinib in recurrent glioblastoma: Failure as a teacher Neuro-oncol 17 910 911 2015 25964312 10.1093/neuonc/nov086 PMC5762007

[46] Brada M Stenning S Gabe R et al Temozolomide versus procarbazine, lomustine, and vincristine in recurrent high-grade glioma J Clin Oncol 28 4601 4608 2010 20855843 10.1200/JCO.2009.27.1932

[47] Buckner JC Shaw EG Pugh SL et al Radiation plus procarbazine, CCNU, and vincristine in low-grade glioma N Engl J Med 374 1344 1355 2016 27050206 10.1056/NEJMoa1500925 PMC5170873

[48] Hardwick SA Deveson IW Mercer TR Reference standards for next-generation sequencing Nat Rev Genet 18 473 484 2017 28626224 10.1038/nrg.2017.44

[49] Yu MK Kramer M Dutkowski J et al Translation of genotype to phenotype by a hierarchy of cell subsystems Cell Syst 2 77 88 2016 26949740 10.1016/j.cels.2016.02.003 PMC4772745

